# P-500. Impact of Antenatal Biologic Therapy on Infant Infection Risk and Vaccination Adherence: A National Cohort Study

**DOI:** 10.1093/ofid/ofaf695.715

**Published:** 2026-01-11

**Authors:** Yoav Kalron, Guy Hazan, David Greenberg, Ilan Youngster, Dana Danino

**Affiliations:** Ben Gurion University, Beer Sheva, HaDarom, Israel; Soroka University Medical Center, Pediatric Department D, Beer Sheva, HaDarom, Israel; Soroka University Medical Center, Pediatric Infectious Disease Unit, Beer Sheva, HaDarom, Israel; Shamir Medical Center, Zerifin, HaMerkaz, Israel; Soroka University Medical Center, Pediatric Infectious Disease Unit, Beer Sheva, HaDarom, Israel

## Abstract

**Background:**

The use of biologic therapies among women of reproductive age has significantly increased, with accumulating evidence supporting their safety during pregnancy. Nevertheless, the long-term implications for the infant's developing immune system remain inadequately understood. This study investigated the association between antenatal exposure to biologic agents and the risk of infections in infants during their first year of life.

**Figure:**
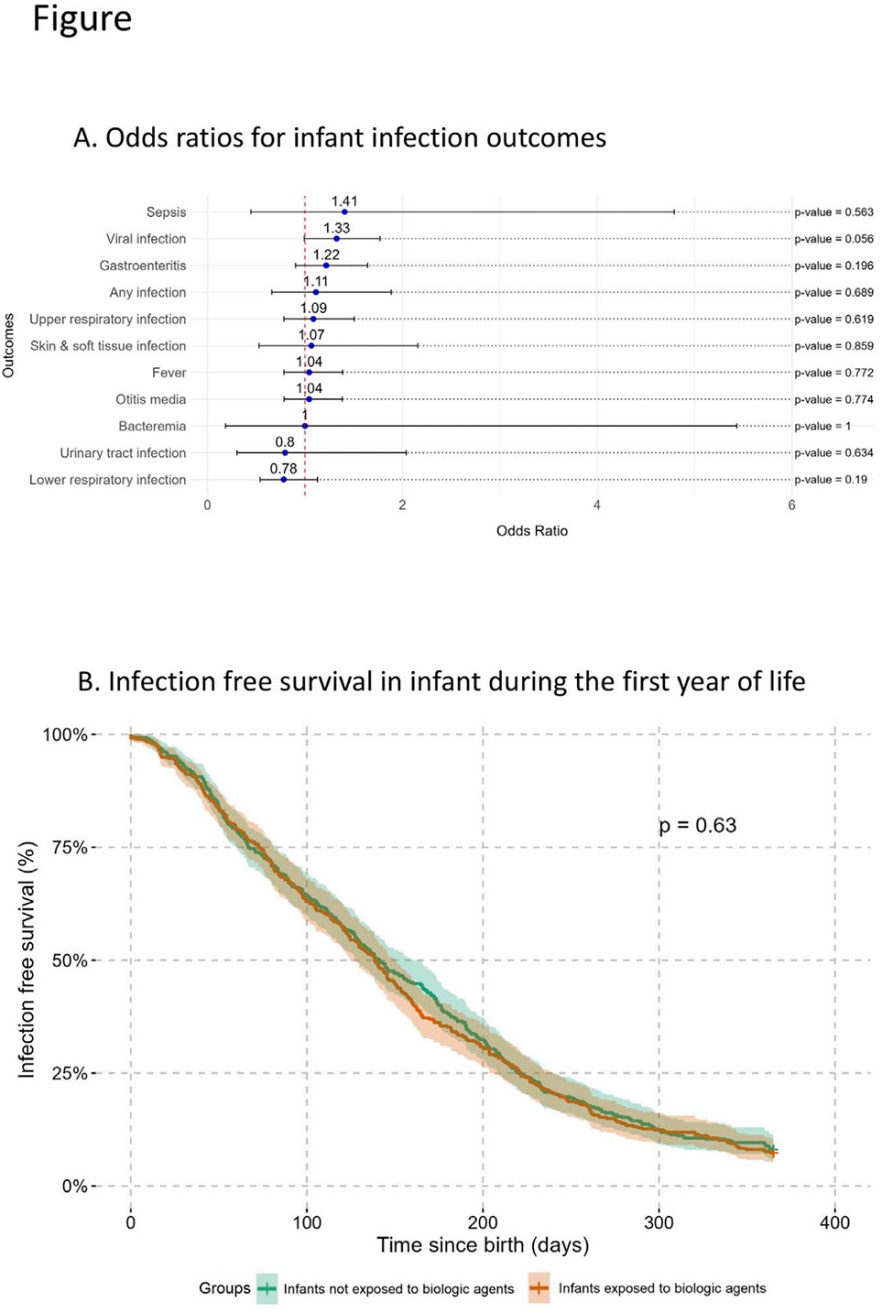

**Methods:**

This nationwide cohort study included all women insured by Clalit HMO who delivered between January 2012 and October 2023. Infants exposed to biologic agents during the second or third trimester were compared to those with no exposure. Propensity score analysis adjusted for maternal chronic medical conditions, pregnancy and delivery characteristics, vaccination status, and timing of birth. Cox regression modeling evaluated the dynamic effects of biologic exposure post-delivery. Infection events were identified using ICD-10 codes. Vaccination adherence was assessed by the total number of vaccine doses administered.

**Results:**

Of 297,480 live births, 395 infants were exposed to biologic therapies antenatally. Infection rates were not significantly higher in infants exposed to biologics compared with unexposed infants (propensity-adjusted odds ratio [OR]: 1.11; 95% CI: 0.66–1.89; *p* = 0.7) (Figure A). Cox regression analysis similarly showed no significant increase in the hazard of infections (hazard ratio: 1.04; 95% CI: 0.90–1.20; *p* = 0.6) (Figure B). Antibiotic consumption and hospitalization rates did not differ significantly between exposed and unexposed infants (OR for antibiotic consumption: 1.22; 95% CI: 0.93–1.62; *p* = 0.2; OR for hospitalization: 1.09; 95% CI: 0.49–2.44; *p* = 0.8). Vaccination adherence was lower for the live-attenuated rotavirus vaccine among biologic-exposed infants (*p* < 0.001), whereas adherence to pneumococcal vaccination was comparable between groups.

**Conclusion:**

Infants exposed to maternal biologic therapy during the second or third trimester of pregnancy did not have an increased risk of infections in the first year of life. However, biologic-exposed infants had significantly lower adherence to the live-attenuated rotavirus vaccine compared to unexposed infants.

**Disclosures:**

David Greenberg, Professor MD, GSK: Advisor/Consultant|GSK: Honoraria|MSD: Advisor/Consultant|MSD: Grant/Research Support|MSD: Honoraria|Pfizer: Advisor/Consultant|Pfizer: Honoraria Dana Danino, Dr. MD, Pfizer: Grant/Research Support

